# Influence of Overloading on Residual Stress Distribution in Surface-Treated Wire Arc Additive-Manufactured Steel Specimens

**DOI:** 10.3390/ma18071551

**Published:** 2025-03-29

**Authors:** Fraser O’Neill, Emmet McLaughlin, Anna Ermakova, Ali Mehmanparast

**Affiliations:** 1 Department of Naval Architecture, Ocean and Marine Engineering, University of Strathclyde, Glasgow G1 1XQ, UK; fraser.o-neill@strath.ac.uk (F.O.); emmet.mclaughlin@strath.ac.uk (E.M.); 2 School of Electrical, Electronic and Mechanical Engineering, University of Bristol, Bristol BS8 1TL, UK; anna.ermakova@bristol.ac.uk

**Keywords:** WAAM, residual stress, neutron diffraction, fatigue, structural integrity

## Abstract

Many countries around the world are in a race against time to decarbonise their energy systems. One of the avenues being explored in detail is Offshore Renewable Energy (ORE), with technologies such as wind, wave, and tidal. All of these technologies are in their infancy within the marine environment and required heavy Research and Development (R&D) to make them commercially viable. With so much demand for these industries, the supply chain is heavily constrained. A solution that has shown great potential to alleviate the pressure on the supply chain is the use of Wire Arc Additive Manufacturing (WAAM) for the use of onsite repair or manufacture for components. This is due to its ability to produce large-scale parts, with low emissions and at a lower cost than other Additive Manufacturing (AM) processes. The opportunity to use this technology could result in shorter downtimes and lead to a reduction in the Levelised Cost of Energy (LCOE). However, knowing that offshore structures are subject to cyclic loading conditions during their operational lifespan, fatigue properties of new materials and manufacturing processes must be well documented and studied to avoid any catastrophic failures. An issue often seen with WAAM is the presence of residual stresses. This study looks at fatigue cracking on Compact Tension C(T) specimens that have undergone laser shock peening and rolling, surface treatment processes that form compressive residual stresses at the surface of the material. In this study, the influence of fatigue overloading on the residual stress distribution in surface-treated WAAM specimens is evaluated and the effectiveness of the post-processing techniques on the subsequent fatigue behaviour is explored.

## 1. Introduction

Overloading within the offshore environment can lead to catastrophic results. The Alexander L. Kielland disaster in the North Sea on the 27 March 1980 is a prime example of this. A fatigue failure on a key bracing member led to the subsequent overloading of other bracing members. This resulted in the platform capsizing and the devastating loss of 123 lives [1]. Since then, the effect of overloading on offshore structures has been an area of concern. Researchers have carried out studies, such as Naderi et al. who looked at the effects of overloading on a jacket structure manufactured from K joints [2]. Other researchers, such as Alibrandi et al. and Martinez-Luengo et al., have looked at creating decision frameworks and analysed the optimal locations for the implementation of Structural Health Monitoring (SHM) systems to monitor fatigue and overloading within these structures [3,4]. All these studies require the use of material properties to understand the fatigue life and effect of overloading on the structure. Liang et al. conducted a study on the Fatigue Crack Growth Rate (FCGR) on S355J2W+N steel, which is commonly used offshore, when subject to overloading, underloading, and a combination of both. The results showed that when the specimen was overloaded in tension, compressive residual stresses were introduced around the crack tip. This led to a decrease in the effective Stress Intensity Factor (SIF) and ultimately the retardation of crack growth, leading to longer fatigue life [5]. Shakeri et al. came to a similar conclusion when looking at welded aluminium plates with AA5083 as the base metal and AA5183 as the weld metal. From FCGR experiments with an overload cycle, it was found that within the weld metal, the compressive residual stresses induced led to the slower FCGR [6]. Grönlund et al. investigated the impact of overloading on longitudinal double-sided fillet welded joints manufactured from S355, S700, and S1100. From this, they found that overloading altered the fatigue life of all three materials due to the relaxation of high residual tensile stresses and the introduction of compressive residual stresses [7].

Wang et al. undertook an analysis of the fatigue crack growth behaviour of AH36 high-strength steel, often used in the marine environment. Using C(T) specimens, they looked at the effect of 1.5, 2.0, and 2.5 overload ratios on the material. From this, it was found that overloading leads to an increase in the fatigue life of the specimens by factors of 1.03, 1.118, and 2.42, respectively [8]. This highlights the large impact overloading can have on the material response. Maljaars et al. conducted a study on the FGCRs of thick-walled K welded plates of S355G8+M using Elgacore DWA55L filler wire. Single overload cycles were applied to the specimens at three stages throughout the crack growth. They concluded that the overloading resulted in a retardation on the FGCR. They also compared overloading models to assess accuracy. They found that the Willenborg model yields good predictions for single overloads but does not cope well with random sequencing of loads [9]. This was a similar conclusion to that of Zhang et al., who investigated the influences of unordered and ordered load spectra for High-Strength Low-Alloy (HSLA) steel for offshore applications. They performed FGCR experiments as per ASTM E647 on Eccentrically loaded Single-Edge Tension, ESE(T), specimens manufactured from DNV F460. The specimens were subjected to four different load spectra and compared to simulation results. For these, Wheeler and Willenborg models were used. These results showed that the Wheeler and Willenborg models struggled to match the experimental results [10]. From these studies, it is clear that introducing residual stresses through an uncharacteristically high load can have a significant impact on the material response and the subsequent crack propagation rates.

As the development of Offshore Renewable Energy (ORE) becomes more prevalent, it is important to consider new emerging technologies to aid in the fast-paced progress towards Net Zero. One of these potential technologies is Additive Manufacturing (AM). This is the process of creating a part via a layer-by-layer process building up to a near-net shape for the desired product. There are many forms of AM, especially for plastic materials. However, in terms of metallic processes, these occur either through a powder or wire-based feedstock. Laser-Powder Bed Fusion (L-PBF) is a common process, but this comes at the cost of having a limited build size and hence rules it out for the use of structural components. Powder Directed Energy Deposition (P-DED) has the advantage of being able to manufacture larger components compared to PBF, making it a more likely choice for the use of structural parts. However, P-DED comes with its drawbacks. This includes its porosity, which can cause stress concentrations within the part, and high material costs due to the feedstock. Wire Arc Additive Manufacturing (WAAM) is similar to P-DED but instead uses a wire feedstock and an arc power source. This process has been shown to have a lower porosity and lower costs associated with the feedstock [11]. Therefore, WAAM is currently the most promising AM method for offshore wind due to the large build volume, short leads, lower cost, and better bulk material quality. This process has the potential to be used as a method for creating replacement parts, coatings, or repairs in short lead times [12,13,14]. This is a benefit for developers as downtime in farms leads to significant losses in revenue. As the supply chain is currently constrained, the use of AM can increase this pressure, resulting in shorter downtimes for structural repairs [15]. The effects of overloading on AM parts are generally not studied. However, an area of great interest concerns the effects of residual stresses on the fatigue life of WAAM-built materials. These stresses are caused by the layer-by-layer process inducing these stresses as a result of thermal cycling. Ganesan et al. discusses the impact of the thermal gradient on the residual stress pattern during manufacturing. The study looks at using ER70S-6 to evaluate induction heating to pre-heat the substrate and each layer prior to deposition. X-ray diffraction was utilised to assess the residual stress pattern. It was found that by using pre-heating, the residual stresses within the material can be reduced by almost 50%, highlighting the sensitive relationship between manufacturing processes and residual stress distribution [16]. Ruan et al. created a WAAM wall made of AM35, which was deposited on a substrate of S355. The study concentrates on the residual stress patterns occurring due to the influence of a flexible clamping boundary condition and a rigid boundary condition using a thick substrate. The redistribution of stresses after cooling and substrate removal was evaluated. This was performed through a mixture of Finite Element (FE) modelling, strain gauges, and sequentially coupled thermal-stress analysis. The conclusions drawn from the investigation highlight that through a clamping method, high tensile stresses were noted in the substrate, with high compressive stresses at the top of the wall. This was thought to be a result of the bending deflection from removing the clamps. When looking at the thick substrate, it was found that the residual stresses were lower in magnitude. After the substrate is removed, all models and boundary conditions exhibit an increase in residual stress at the bottom of the wall. The work highlights the need for careful planning in the manufacture of components to ensure residual stresses do not negatively impact the operational life of the component [17].

Dahaghin et al. investigated using ER70S-6 wire with WAAM to arrest crack growth from a steel plate made from S355J2+N with a central notch. They conducted fatigue testing on a notched only specimen, a notched specimen with as-built WAAM material deposited in a cubic form, and a notched specimen with WAAM material deposited over the notch then machined to a pyramid shape. The fatigue testing showed that the machined WAAM yielded the best results, indicating an infinite fatigue life for the stress range. The as-built repair yielded a better fatigue life than the specimen without repair. However, the crack initiated at the interface between the WAAM material and the plate. Thermo-mechanical and Inherent Strain Method (ISM) FE modelling was employed to evaluate the residual stresses within the parts. The results showed that residual stresses within the interface region between the WAAM and substrate produced the highest stress. The as-built specimen showed these at the location of cracking in the experiment. Several shapes and manufacturing parameters were evaluated and it was found that by applying a pyramid shape machining strategy, the residual stresses at the interface can be reduced [18]. This highlights the effectiveness of WAAM as a repair solution, while also showcasing the need for proper design considerations to be made in order to maximise the fatigue life of components through a reduction in residual stresses. Ermakova et al. looked at the residual stress pattern of WAAM ER70S-6 and ER100S-1 after undergoing rolling and laser shock peening surface treatments. From this study, it was shown that by using surface treatments such as laser shock peening, it was possible to induce compressive residual stresses at the surface, resulting in a longer fatigue life. The same could not be said for the rolled specimens, with the ER100S-1 specimen experiencing a lower fatigue life than the untreated specimen, again showing the importance of understanding the impact of residual stresses in WAAM [19]. This paper looks to follow on from this study by looking at the residual stress distribution after fatigue cracking and the introduction of an overload during a cyclic loading condition. This was achieved through subjecting the same samples that were used in the previous study for fatigue cyclic loading at a range that would induce overloading. Neutron diffraction was then conducted, comparing results to the previous study. The objective of this paper is to highlight the change in residual stress pattern of surface-treated WAAM specimens. The hope is that these results will provide further insights to evaluate the suitability of WAAM for offshore environments and enable more modelling of WAAM components that undergo both surface treatment and fatigue.

## 2. Methodology

### 2.1. Manufacturing

A wall of WAAM built material was manufactured through Cold Metal Transfer (CMT), using ER100S-1 welding wire as the feedstock, creating a wall approximately 24 mm thick. The manufacturing process included utilising a programmed robotic arm, a CMT power source, and a torch. Within the system, shielding gas and wire were supplied throughout. The manufacturing parameters and chemical composition of the ER100S-1 wire used can be seen in Table 1 and Table 2, respectively. A previous study by Ermakova et al. conducted a microstructural and mechanical investigation into the properties of ER100S-1 material and can provide more insight into the material [20]. The building strategy employed was an oscillation method aimed at reducing the presence of columnar grains. The wall was constructed onto a substrate of EN10025 rolled structural steel. The substrate was clamped to the bench via eight clamps. This was performed to reduce the distortion within the build plate during the high temperatures experienced in manufacturing. Once the wall reached ambient temperature, the clamps were removed, and the wall was detached from the substrate. Electro Discharge Machining (EDM) was used to extract two notched Compat Tension, C(T), specimens from the wall. The orientation of the crack path is vertical, i.e., the crack path is perpendicular to the AM layer, with one specimen coming from the top of the wall and the other coming from the bottom. The C(T) specimens were designed to the ASTM E647 standard with the following dimensions: width, W = 50 mm; height, H = 60 mm; total thickness, B = 16 mm, and initial crack length, *a*_0_ = 17 mm. Clip gauge knife edges were also machined on the specimens as per the ASTM 1820 standard for conducting fatigue crack growth tests.

### 2.2. Surface Treatment

One C(T) specimen was subjected to high-pressure rolling. The rolling parameters used were derived from the Finite Element Analysis conducted by Pi et al. [21]. As a result, a 20 mm wide high-strength steel roller with a 50 mm diameter was used. The rolling was applied from the crack tip of the first C(T) specimen for a total distance of 8 mm at a radial rate of 100 mm/s at an 80 kN load. The specimen was clamped to the bench via three clamps, a roller was attached to a movable rig, and a laser tag was used for alignment. The adoption of 8 mm was based on Pi et al. [21] as their study demonstrated that it resulted in the optimal residual stress pattern. The rolling was performed sequentially on both free surfaces. This method developed an area with approximate dimensions of 20 × 8 mm^2^ for the rolled surface ahead of the specimen’s V-notch.

The other C(T) specimen was surface treated through a laser shock peening method for the same area of 20 × 8 mm^2^ around the machined V-notch tip, applied on both sides of the specimen so that it could be comparable to the rolled specimen. The laser parameters used for this were a spot size of 3 mm^2^, an energy level of 8.1, a power density of *J* = 5 GW/cm^2^, and a pulse duration of 18 ns. Three layers were applied, thus ensuring 300% coverage. More information about the surface treatment processes can be found in [19].

### 2.3. Fatigue Cracking

**Figure 1:**
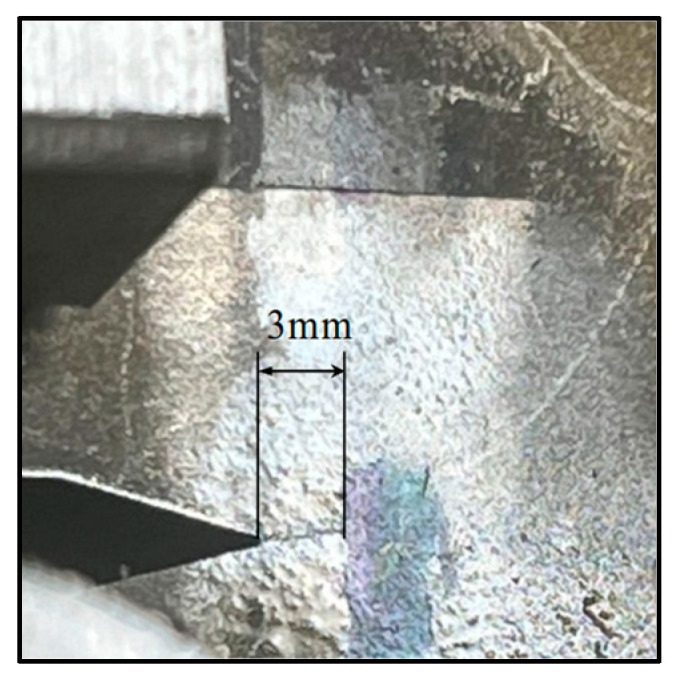
High-resolution image of 3 mm fatigue crack growth on C(T) specimen.

Both C(T) specimens showed fatigue cracking of 3 mm from the machined V-notch tip through an applied cyclic tensile load, as shown in Figure 1. This was followed on from the distribution of residual stress analysis work carried out by Ermakova et al. [19] looking at the impact of fatigue on the material and evaluating how the surface treatments affect this distribution. The fatigue testing was implemented via constant amplitude loading, with R = 0.1 and a maximum load of 18 kN on an Instron 8800 servo-hydraulic system. High-resolution cameras were mounted on both sides of the specimens to monitor the crack growth and terminate the test at the desired crack length.

### 2.4. Neutron Diffraction

**Figure 2:**
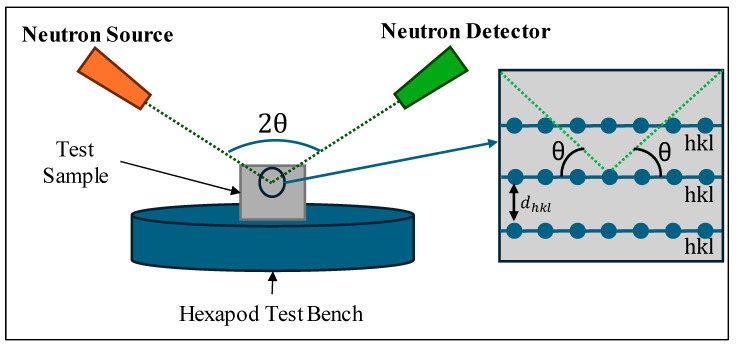
Neutron diffraction method diagram.

Neutron diffraction is a technique used in the imaging and analysis of crystal materials and their properties. This method works off the principles of Bragg’s law, shown in Equation (1), whereby neutrons are fired from a detector to a specified point within a material. The neutrons diffract off internal planes within the structure of the material and are captured by a detector where the diffraction angle can be measured. Figure 2 showcases a simple diagram for this process. The diffraction angles, $\theta^{hkl},$ are calculated along with the wavelength of the neutron beam; λ can then be used to calculate the interplanar spacing, $d^{hkl}$, and subsequent internal strains. This method was utilised at the Institut Laue-Langevin (ILL) using their advanced neutron strain analyser, SALSA.

(1) $$2d^{hkl}\sin\left(\theta^{hkl}\right)=\lambda$$

**Figure 3:**
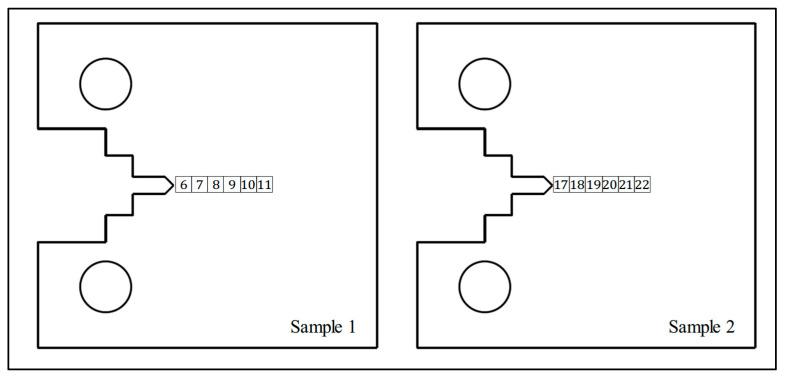
Location and labelling of *d*_0_ reference cubes in specimens 1 (laser-shock-peened C(T) specimen) and specimen 2 (rolled C(T) specimen).

In order to account for the material variability in the WAAM built specimens, reference strain-free cubes were extracted using EDM from a nominally identical specimen which had not undergone any surface treatment and can be seen in Figure 3 along with their extraction location. As a result of this and due to their size of 5 × 5 × 5 mm^3^, it is assumed that no residual stress is present in the cubes. Interplanar spacing was measured in these cubes for use as a reference distance of the unstressed material; these will be referred to as *d*_0_ cubes for the rest of the paper.

**Figure 4:**
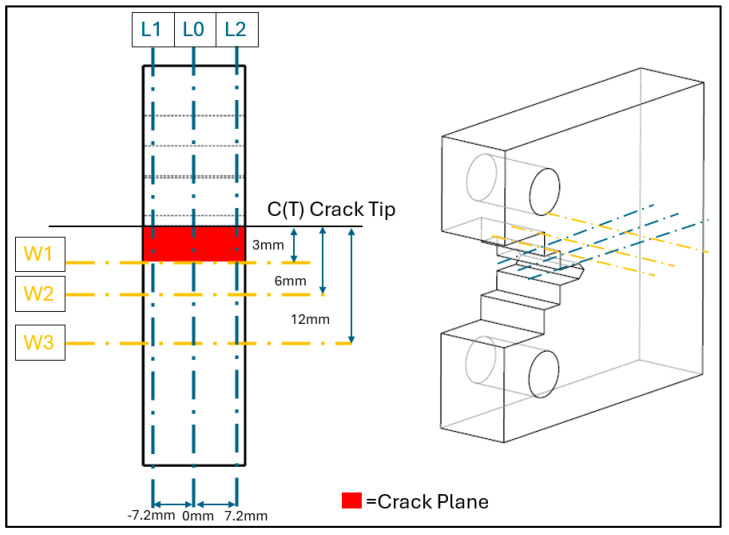
Neutron diffraction scanning paths for the specimens.

The raw data were processed on ILL’s internal software LAMP Version 23 [22]. Here, the data were integrated to obtain diffractograms and peak fitted using a Gaussian function and linear background. From these data, interplanar spacing was calculated, based on Equation (1), along three paths along the C(T) specimens and at three through thicknesses, which are depicted in Figure 4. These interplanar spacings were used to calculate the strain, $\varepsilon^{hkl},$ at each of the points, using Equation (2) and the respective *d*_0_ valve at that point location within the specimen. With these strains and material property values—of Young’s modulus, $E_{hkl}$, and Poisson Ratio, $\upsilon_{hkl}$—from a previous study by Ermakova et al. [19], residual stresses, $\sigma_{yy}$, were calculated throughout the C(T) specimen, as per Equation (3).

(2) $$\varepsilon^{hkl}\frac{\sin\theta_{0}^{hkl}}{\sin\theta^{hkl}}-1$$

(3) $$\sigma_{yy}=\frac{E_{hkl}}{\left(1+\upsilon_{hkl}\right)\left(1-2\upsilon_{hkl}\right)}[\left(1-\upsilon_{hkl}\right)\varepsilon_{yy}+\upsilon_{hkl}\left(\varepsilon_{xx}+\varepsilon_{zz}\right)]$$

### 2.5. Numerical Modelling

#### 2.5.1. Finite Element Analysis Method

To test the hypothesis that inducing high stresses to crack the specimen will result in a residual stress pattern akin to overloading, numerical modelling was conducted on a simplified representation of the specimen. As evidenced by Bouchard et al. [23], an effective understanding of the residual stress patterns can severely influence the rate of crack growth and time taken for a crack to initiate. It is also explained in [23] that the residual stress patterns discussed were for defects without stress relieving. Studies, such as that performed by Noghabi et al. [24], have shown that initial residual stresses can decrease rapidly after a few fatigue cycles, and stated that the load amplitude has a great influence on the reduction in residual stresses. The purpose of the simulations is to back up the assumption that an adequate load applied to the C(T) specimen will result in a stress pattern that would be seen if the specimen were overloaded.

Two factors make effective and accurate modelling of the supposed issue a difficult task and result in major uncertainties. The first of which, the variability of the residual stress distribution from surface treatment, is difficult to accurately replicate for both surface treatment methods, and the other is the added complexity that additive manufacturing and WAAM brings.

Using FEA to accurately model and simulate the WAAM process has proven difficult for a variety of reasons. The process contains a plethora of physical transformations, such as in thermodynamics, phase transitions, and solidification. Combining this with the complex chemical, mechanical, and metallurgical phenomena results in reduced confidence in modelling techniques [25]. Layer-wise deposition and thermal history can result in complex thermal cycles inducing residual stress patterns, of which there is not a generally accepted model for their prediction. The layer-by-layer approach also induces a great deal of anisotropy behaviours, changing the properties in all directions and resulting in a great deal of variation according to location. Furthermore, the process is particularly sensitive to minor changes in environment or equipment, as minor deviations can amplify over the extended manufacturing period and lead to significant inaccuracies [26].

Compounding the modelling concerns are the difficulties in achieving accurate and useable residual stress patterns in computational modelling for surface-treated components. Variability in surface treatment processes can have significant consequences in the location and magnitude of the residual stress. Laser shock peening is highly sensitive to intensity, duration, and coverage [27], whereas in rolling, the roller size, pressure, and sequence has the same effect. The complex response of the material must also be accounted for, especially when dealing with the complexities of a WAAM-made component. The non-linear response and pre-existing residual stresses may compound, and reduce or increase the effectiveness of the surface treatment procedures.

**Figure 5:**
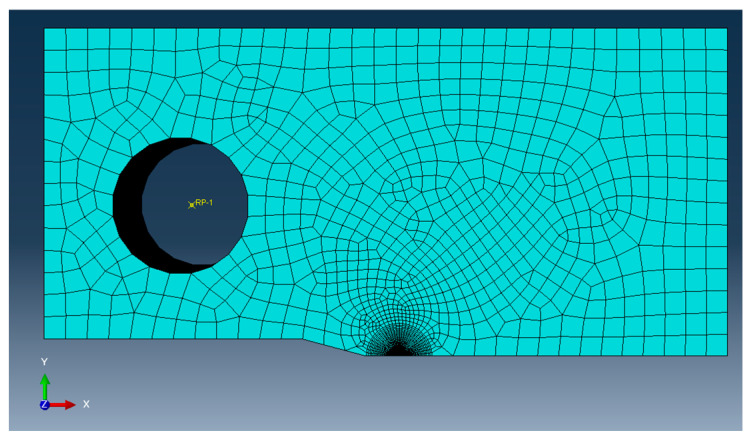
Finite element model used for residual stress prediction.

**Figure 6:**
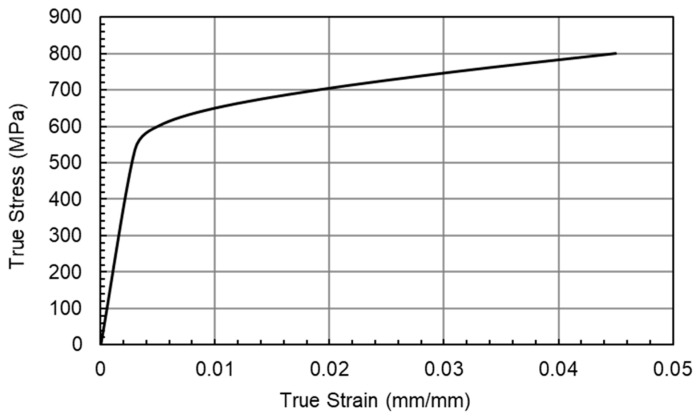
True stress–strain response of ER100S-1, taken from [20], as used in FEA simulations.

Combining the variable nature of WAAM with the variations in residual stresses due to surface treatment increases the potential variances within the model and real-world testing. For these reasons, the numerical modelling was completed using the assumption of homogenous material properties, with no surface treatment and assuming no initial residual stresses. To check if the load of 18 kN was sufficient to cause overloading, Finite Element Analysis (FEA) was conducted on the final loading cycle, to model any residual stress effects present due to the fatigue cracking process. The model was completed in the FEA software package ABAQUS 2022 (version 6.14-2), and a standard explicit solver was utilised. The model was constructed with the dimensions discussed above, and to improve computational efficiency, a horizontal symmetry boundary condition was introduced along the centre of the specimen (crack path), as shown in Figure 5. A wedge-type circular mesh was implemented at the crack tip with a radius of 0.05 mm, with 30 elements along half the circumference. The surrounding area was made up of a hex mesh. The cross-span of the specimen was split into 64 elements, leading to a total number of elements of 135,774. A load of 18 kN was applied to a reference point in the centre of the pin, with a fixed connection to the surface of the pin hole, ensuring the elements around the pin did not deform. The von Mises criterion was used to identify the yield surface, and for ease of simulation, isotropic material behaviour was assumed. The elastic modulus was recorded as 180 GPa, Poisson’s ratio as 0.3, and the stress–strain response as shown in Figure 6. These material properties are for ER100S-1 WAAM-built steel and were taken from Ermakova et al.’s investigation into the mechanical properties of WAAM-built low-carbon steel [20].

#### 2.5.2. Analytical Solution

To further investigate the accuracy of the hypothesis, analytical methods were used to estimate a plastic zone radius along the crack tip. The modified Irwins equation (Equation (4)) was used to predict the radius of the plastic zone, *r_p_*, in the crack path [28].

(4) $$r_{p}=\frac{1}{2\beta \pi }{\left(\frac{K_{I}}{\sigma_{y}}\right)}^{2}$$

In the equation above, β is a parameter utilised to identify where in the material the plastic zone is located. Near the free surface of the specimen the plane stress condition is often considered as β=1. However, in the bulk of the material, it is usually considered as a plane strain condition—thus the plastic zone will be reduced— and therefore β=3. The yield point of the material $\sigma_{Y}$ is found through Figure 6, which for ER100-S can be taken as 583 MPa, and finally the stress intensity factor $K_{I}$ can be found through the following equation:

(5) $$K_{i}=\frac{P_{i}}{{\left(BB_{N}W\right)}^{0.5}}\times f\left(a_{i}/W\right)$$

where $a_{i}$ is the instantaneous crack length, *B* is the thickness, *B_N_* is the net thickness between the side grooves, *W* is the width, *P_i_* is the incremental load, and $f\left(\frac{a_{i}}{W}\right)$ is given by Equation (6).

(6) $$f\left(\frac{a_{i}}{W}\right)=\frac{2+\frac{a_{i}}{W}}{{\left(1-\frac{a_{i}}{W}\right)}^{1.5}}\left[0.886+4.64\frac{a_{i}}{W}-13.32{\left(\frac{a_{i}}{W}\right)}^{2}+14.72{\left(\frac{a_{i}}{W}\right)}^{3}-5.6{\left(\frac{a_{i}}{W}\right)}^{4}\right]$$

## 3. Results

### 3.1. Finite Element Analysis Results

**Figure 7:**
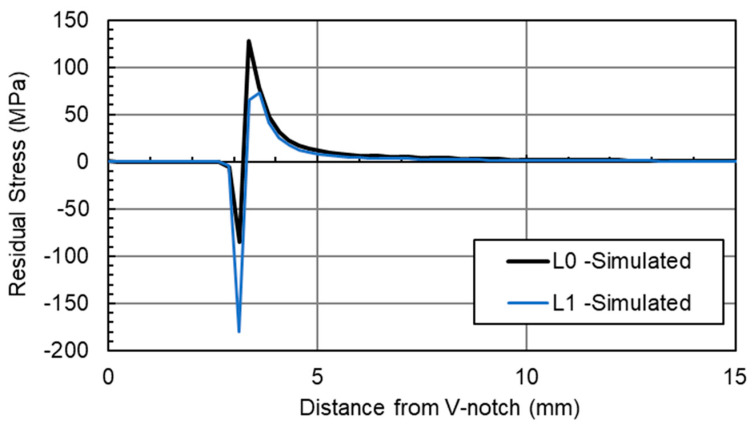
Simulated residual stresses due to overload in the cracking process.

FEA was carried out to investigate the hypothesis that the fatigue cracking procedure at high load would result in a residual stress pattern similar to overloading. Figure 7 shows the stresses along the L-lines of the specimen, which run parallel to the crack path, along the midpoint of the thickness (L_0_), and ±7.2 mm from the entre plane (L_1_ and L_2_), as shown in Figure 4. These L-lines allow a direct comparison from the simulation to the recorded results from neutron diffraction testing. A plane stress condition was employed in FEA simulations to predict residual stresses along L_1_ and L_2_, while a plane strain condition was applied for predictions along L_0_. The results in Figure 7 show that in both the centre plane (where plane strain conditions dominate) and the near surface (plane stress conditions), a compressive residual stress around the crack tip is visible, followed by a tensile residual stress levelling off, becoming stress balanced further away from the crack tip. This is indicative of overloading, meaning residual stresses are likely to have been introduced, because of the fatigue cracking procedure [29]. L_0_ represents the midpoint of the material, and therefore the material is operating in conditions akin to plane strain, in comparison to the near surface (L_1_ lines), where plane stress conditions dominate.

The recorded values of maximum and minimum residual stresses are not directly comparable to what is to be expected with the previously mentioned limitations of the model, and for the potential calibration errors within the neutron diffraction test procedure. In regard to the size of the plastic zone, the FEA model was backed up by an analytical solution. From Equations (4)–(6), the plastic zone size in plane strain conditions can be estimated at 0.63 mm in the plane stress condition (near surface) and 0.21 mm in the plain strain condition.

### 3.2. Laser-Shock-Peened Specimen Results

#### 3.2.1. Laser-Shock-Peened Specimen Crack Path Results

**Figure 8:**
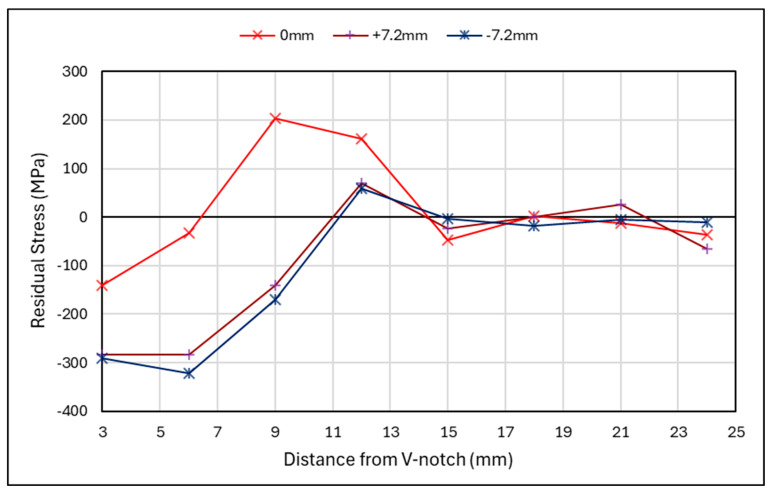
Residual stress along crack path direction for shot-peened specimen.

Figure 8 details the neutron-diffraction residual stress measurements along the crack path direction of the laser-shock-peened C(T) specimen at the middle section, and near both surfaces at 7.2 mm from the centreline. Regarding the shape of the graph seen in Figure 8, the initial shape at and ahead of the crack plane shows indications that overloading has taken place. This is due to the presence of high compressive stresses at the crack front followed by high tensile stress, indicating the presence of a plastic zone. This behaviour has been noted by researchers such as Lam et al. who studied the plastic zone of CoCrFeMnNi after fatigue and overload via neutron diffraction measurements [30]. The higher compressive stress at the near surface may be a result of the plane stress condition occurring at the surface.

#### 3.2.2. Laser-Shock-Peened Specimen Through-Thickness Results

**Figure 9:**
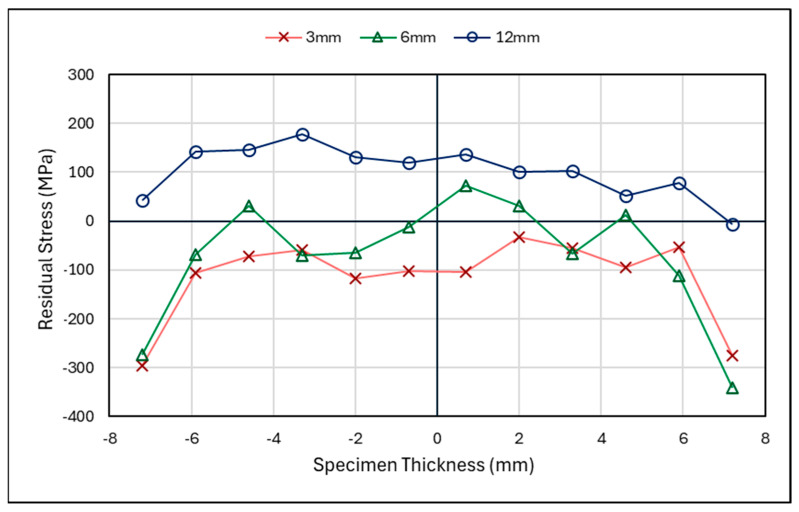
Laser-shock-peened specimen—residual stress versus specimen thickness.

In Figure 9, the neutron-diffraction residual stress measurements through the thickness of the C(T) specimen after shock peening and fatigue cracking are shown, with measurements at 3, 6, and 12 mm from the V-notch tip. Across the crack front at 3 mm and ahead of the crack front at 6 mm, high compressive stresses can be seen, which is further indication of overloading. The higher compressive stresses noted at 3 and 6 mm highlighted the effectiveness of the laser shock peening at creating compressive residual stresses at the surface when compared to the 12 mm line, which did not undergo peening. Pavan et al. have reported similar residual stress patterns for through-thickness measurements of laser-shock-peened material that are similar to those at 3 mm and 6 mm [31]. This highlights that laser shock peening has been successful in this region and that the residual stress at 12 mm is a result of the as-built conditions and loading.

### 3.3. Rolled Specimen Results

#### 3.3.1. Rolled Specimen Crack Path Results

**Figure 10:**
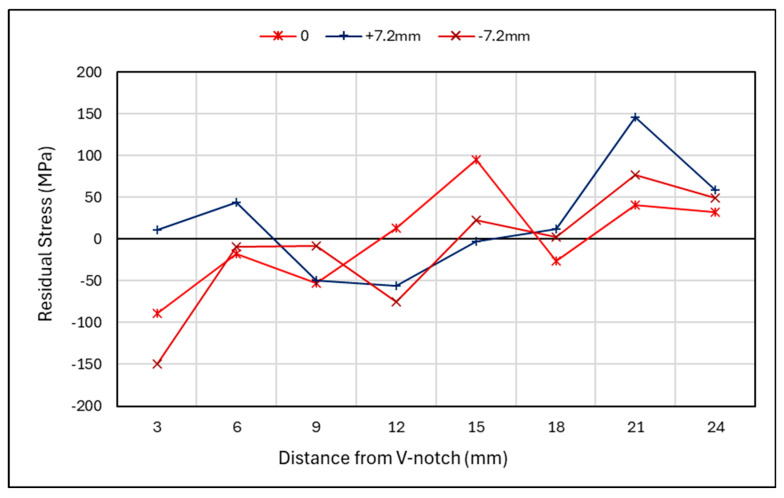
Rolled specimen—residual stress along crack path direction.

Figure 10 shows the residual stress along the crack path direction for the rolled specimen, for the centreline section and near the surfaces at both ±7.2 mm from the centreline. The stresses after the rolled section, >8 mm from the V-notch, show a similar trend throughout the specimen. However, for the rolled section, an uneven distribution can be seen throughout the material’s thickness, with 0 mm and −7.2 mm lines showing similar trends in compressive stress, which start to increase after 3 mm to tensile stresses. This can be an indication of overloading. This observation cannot be said for the +7.2 mm line, which shows tensile stresses are present initially before becoming compressive and then increasing to tensile stresses. This could be due to the sequential rolling process leading to an uneven distribution of residual stress, or the result of a non-uniform distribution of residual stress caused by the thermal cycles of the WAAM process.

#### 3.3.2. Rolled Specimen Through-Thickness Results

**Figure 11:**
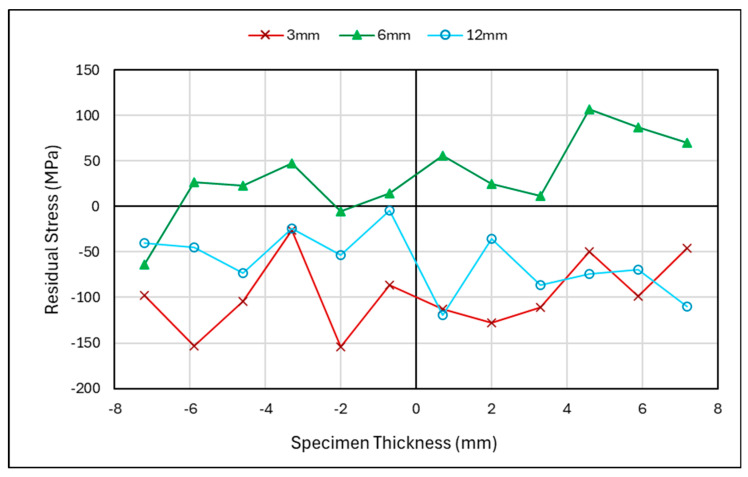
Rolled specimen—residual stress versus specimen thickness.

In Figure 11, the residual stresses through the thickness of the C(T) specimen after rolling and cracking are shown, with measurements at 3, 6, and 12 mm from the V-notch tip. These measurements back up the trend in Figure 10 of an uneven stress distribution throughout the thickness of the material, with more compressive stresses noted between −7.2 and 0 mm. These observations confirm the findings detailed in [19] that for the parameters considered in this study, the laser shock peening technique provides more reliable outcomes compared to the rolling method, and that fatigue crack growth retardation through introducing compressive residual stresses is more achievable using the laser peening technique.

## 4. Discussions

### 4.1. Laser-Shock-Peened Specimen

#### 4.1.1. Laser-Shock-Peened Crack Path Measurements

**Figure 12:**
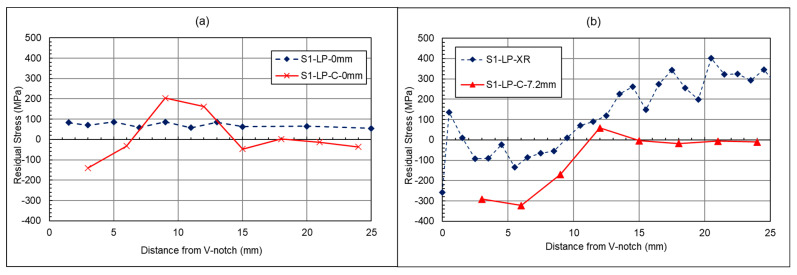
Shot-peened specimen—residual stress measurements along crack direction at (**a**) middle thickness and (**b**) near surface.

The following figures compare results with those of a study conducted by Ermakova et al. [19]. In their study, the same specimens had undergone residual stress measurements before and after surface treatment and in the absence of any fatigue cracks. It should be noted that the residual stress before surface treatment is not discussed in this paper, as further information and details can be found in the previous work of Ermakova et al. [19]. These results are labelled as S1-LP. The results from the present study focus on these specimens after fatigue cracking, with the specimens labelled as S1-LP-C, to showcase the effect of cracking on surface-treatment-induced residual stresses. In Figure 12a, the stress distribution profile has changed in terms of the distribution of the residual stresses throughout the length of the material. The highly compressive stresses at the 3 mm crack front, followed by high tensile stresses at 8 mm, are an indication of the material being overloaded during the fatigue cracking process. In Figure 12b, the cracked specimen has been measured at −7.2 mm from the centre via a neutron diffraction technique, whereas the previous study by Ermakova et al. [19] used an X-Ray diffraction method for near-surface residual stress measurement in surface-treated C(T) specimens. This means the results are not directly comparable due to the different X-ray and neutron diffraction test methods, and their corresponding gauge volumes. However, they both give a suggestion as to what is occurring near the surface of the material. Figure 12b shows a large compressive residual stress immediately after the crack tip, followed by a tensile residual stress and a relatively low residual stress far from the crack tip, consistent with the expected results from overloading the C(T) specimen.

**Figure 13:**
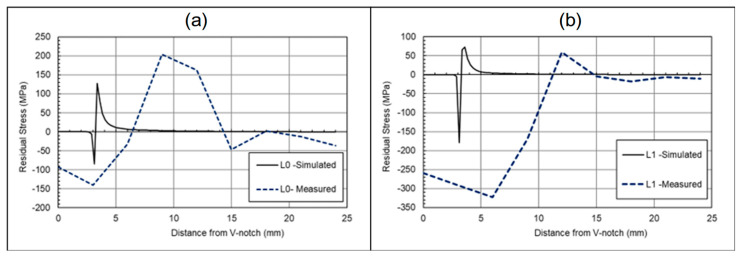
The measured and predicted residual stresses in the longitudinal direction (**a**) at the centre of the through thickness and (**b**) at the near surface.

Comparing the measured values of residual stress with the simulated results, such as in Figure 13, can deepen the understanding of the impact of overloads on the residual stress distribution ahead of the crack tip. As seen in this figure, whilst the maximum residual stresses and size of the plastic zone differ, the patterns are consistent. For the L_0_ lines, a smaller compressive residual stress is expected in comparison to the tensile loads, along with a smaller plastic zone in comparison to the near-surface L_1_ lines. The L_1_ lines also follow a similar pattern in both the simulation and measured data, with a higher compressive load, followed by a lower tensile load. The two main differences from the measured data are the plastic zone radius, and the level of residual stresses, both of which are greater than predicted within the simulated solution. The potential cause of the change may be down to the numerical models assuming a perfectly isotropic material, whilst the WAAM-built components may be anisotropic, and material properties will vary. Moreover, the numerical models in this study were completed without any pre-existing residual stress profiles as a result of surface treatment or residual stresses remaining in the C(T) specimens after deposition and extraction. It is thought that this may have also impacted the stress distribution and the resulting plastic zone prediction; however, the purpose of the FEA simulations was not to predict the residual stress profiles but to understand the impact of tensile overloads on the formation of compressive residual stresses ahead of the crack tip.

#### 4.1.2. Laser-Shock-Peened Through-Thickness Measurements

**Figure 14:**
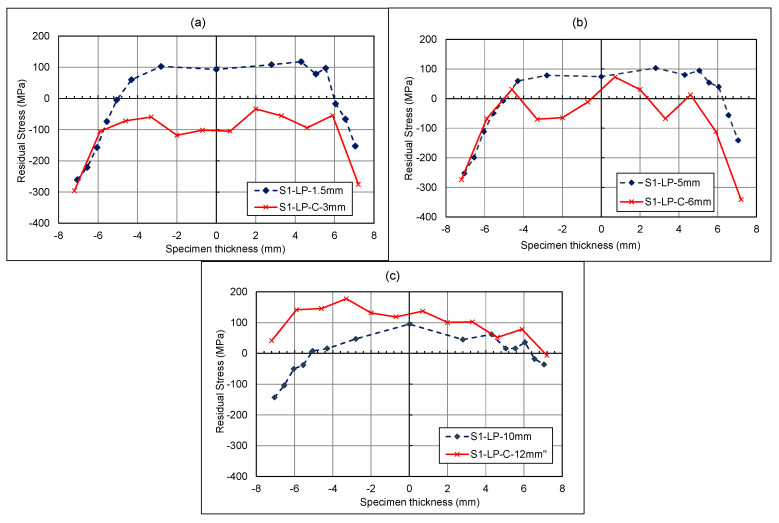
Shot-peened specimen—cracked and uncracked residual stress measurements for through thickness at (**a**) 1.5–3 mm, (**b**) 5–6 mm, and (**c**) 10–12 mm from V-notch Tip.

Figure 14a–c showcase the through-thickness measurements before and after fatigue cracking. From previous work [19] and this study, laser shock peening has been proven to introduce compressive residual stress at the surface and thereafter to counteract tensile residual stress within the centre of the material. However, in Figure 14a,b, the centre remains in the compressive region compared to its state before cracking. Figure 14c, which shows the area past the surface-treated section, presents a higher tensile stress than before cracking. Therefore, with compressive regions at 3 mm and 6 mm, there is evidence of a compressive plastic zone; with a corresponding tensile force at the 12 mm region This behaviour is typical of overloading. Analysis through the thickness and along the specimen length shows that overloading has occurred, inducing a plastic zone ahead of the crack tip and leaving an opposing tensile zone in the residual stress distribution. The impact of the surface treatment is more evident in the through-thickness measurement where the surfaces remain as highly compressive zones. In certain scenarios, the presence of a plastic zone can lead to longer fatigue lives due to the plastic zone retarding the crack growth. This has been proven by other researchers such as He et al., Salvati et al., and Gadallah et al., who have all investigated the impact of an overloading condition on the fatigue crack behaviour of specimens [32,33,34]. To better understand and evaluate the effect of the surface treatment on the plastic zone, a similar experiment should be conducted in future work, looking at the residual stress pattern of a specimen after overloading with no surface treatment.

### 4.2. Rolled Specimen

#### 4.2.1. Rolled Specimen Crack Path Measurements

**Figure 15:**
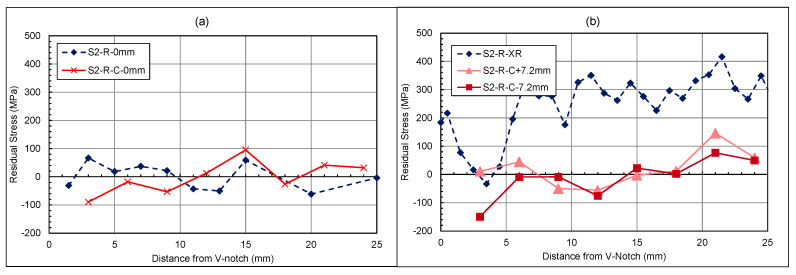
Rolled specimen—cracked and uncracked residual stress measurements along crack direction at (**a**) middle thickness and (**b**) near surface.

When looking at the rolled specimen in Figure 15, a similar overloading trend can be seen for the centre of the specimen at 3 mm along the length of the specimen. This compressive region exhibits an overload behaviour. However, further along the length of the specimen, between 8 mm and 15 mm, there is a large increase from compressive to tensile stress. A similar pattern is noted for before cracking, denoted as S2-R, and after cracking, denoted as S2-R-C, suggesting that it is a result of rolling rather than cracking. This may be due to a plastic zone being created from the rolling procedure, whereby a tensile strain region is present beyond the rolled section. This theory is reinforced by Vinogradov et al., who have conducted a numerical study on the influence of rolling on cold-rolled strips. From this, they found that when rolling is conducted over short distance with high loads, a plastic zone can be induced ahead of the rolled area [35]. The heightened values at 15 mm for the cracked specimen could be a result of the redistribution of stresses. When looking at the surface region in Figure 15b, it shows that before cracking, a compressive stress was induced in the rolled area. However, when looking at the crack front after fatigue cracking, there is a difference in compressive stress from one side to the other. This further highlights the uneven distribution of residual stresses due to sequential rolling. Beyond the rolled region, a redistribution of stress has occurred, with a lower average stress along the length of the specimen once fatigue cracking has occurred.

#### 4.2.2. Rolled Specimen Through-Thickness Measurements

**Figure 16:**
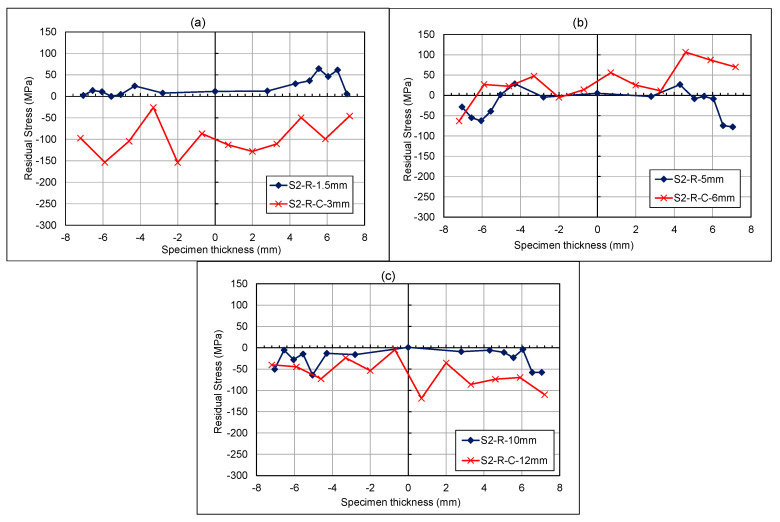
Rolled specimen—cracked and uncracked residual stress measurements for through thickness at (**a**) 1.5–3 mm, (**b**) 5–6 mm, and (**c**) 10–12 mm from V-notch tip.

From the through-thickness measurement results in Figure 16a–c, a notable difference between the specimens can only be observed for the 3 mm region. This is an indication that a small overloading region exists ahead of the crack front, with minor stress redistribution at each stage. The results from this study show that the fatigue overloads can significantly influence the residual stress redistribution pattern in WAAM-built specimens in both longitudinal and through-thickness directions. While the impact of tensile overloads on the formation of compressive residual stress ahead of the crack tip was investigated in this study, further research needs to be carried out in future work to develop accurate numerical prediction models to estimate the redistribution of residual stresses under various levels of overload and in the presence of complex pre-existing residual stress profiles in WAAM-built components.

## 5. Conclusions

This study explored the effect of fatigue overloads on the residual stress distribution in surface-treated specimens made with the Wire Arc Additive Manufacturing (WAAM) technique. C(T) specimens underwent cyclic loading with a load ratio of *R* = 0.1 and maximum load of 18 kN, to achieve 3 mm of fatigue cracks in the specimens. Subsequently, neutron diffraction measurements were carried out to evaluate the residual stress distribution in laser-shock-peened and rolled C(T) specimens. The conclusions of this study are outlined below:

- The rolling procedure not only introduced compressive residual stress in the treated area, but a counteractive tensile stress for the region between 8 and 15 mm, where the region was not rolled.
- Overloading was introduced by fatigue loading, with a relatively high maximum load of 18 kN on the rolled and laser-shock-peened specimens, which resulted in high compressive residual stress profiles at the crack front, and subsequent tensile stresses further away from the crack tip.
- Fatigue cracking redistributed the residual stresses, leading to a reduction in the mean stress for the laser-shock-peened specimen at both the surfaces and the centre of the specimen. However, for the rolled specimen, only the free surface saw a reduced mean stress, whereas the centre did not. This could be due to the presence of the plastic strains created from the rolling procedure.
- The FEA aligns with the overloading theory and provides similar patterns to those obtained from experimental measurements. However, the simplistic nature of the model results in significant variations in the maximum residual stresses predicted from the simulations.
- More complex FEA models can be created in future work to accurately predict the residual stress redistribution in WAAM-built components by implementing material variability and introducing any pre-existing residual stress profiles that might have been previously formed during the deposition process, extraction of specimens, or the surface treatment.

## Figures and Tables

**Table 1:** Manufacturing parameters of CMT of WAAM.

Manufacturing Parameters for CMT of WAAM	
Shielding Gas	AR + 20% CO_2_
Gas Flow Rate	15 L/min
Robot Travelling Speed	7.33 mm/s
Wire Diameter	1.2 mm
Wire Feed Speed	7.5 m/min
Dwell Time	120 s

**Table 2:** Chemical Composition of ER100S-1 WAAM Wire.

Chemical Composition of ER100S-1 WAAM Wire (wt.%)						
	**C**	**Mn**	**Cr**	**Si**	**Ni**	**Mo**
ER100S-1	0.08	1.70	0.20	0.60	1.50	0.5

## Data Availability

The original contributions presented in this study are included in the article. Further inquiries can be directed to the corresponding author.
